# Clinical characteristics and outcomes in 50 children with autoimmune hepatitis: a retrospective study from a single centre in China

**DOI:** 10.3389/fmed.2025.1733006

**Published:** 2026-01-12

**Authors:** Yandi Yang, Fan Yang, Min Yang, Zherui Liu, Shuhong Liu, Linlin Lian, Binxia Chang, Lijun Shen, Hua Tian, Qingsheng Liang, Songhai Chen, Shuna Dong, Chunyu Li, Huan Xie, Yan Zhong, Zhengsheng Zou, Ying Sun, Yun Zhu

**Affiliations:** 1 The 82nd Group Army Hospital of PLA, Baoding, Hebei, China; 2 Hainan Branch, Shanghai Children's Medical Center, School of Medicine, Shanghai Jiao Tong University, Sanya, China; 3 Department of Preventive Medicine, Hubei University of Chinese Medicine, Wuhan, China; 4 Peking University 302 Clinical Medical School, Beijing, China; 5 Department of Pathology and Hepatology, The Fifth Medical Center of PLA General Hospital, Beijing, China; 6 Handan City Infectious Disease Hospital, Handan, Hebei, China; 7 Senior Department of Hepatology, Chinese PLA General Hospital, Beijing, China; 8 The 989 Hospital of the People's Liberation Army Joint Service Support Force, Luoyang, China; 9 Yunnan University, Kunming, China

**Keywords:** autoimmune hepatitis, children, outcome, treatment, type

## Abstract

**Objectives:**

Little is known about autoimmune hepatitis (AIH) in Chinese children. The study aimed to explore the clinical characteristics and predictors of outcomes in the Chinese paediatric AIH cohort.

**Methods:**

A retrospective review of all paediatric AIH cases from 2015 to 2025 was conducted at a single centre in Beijing, China.

**Results:**

Of the 50 enrolled cases, 39 (78.0%) presented with type 1 AIH (consisting of 30 female children (60.0%); median age of 9.2 years). At presentation, 24 (48.0%) patients had cirrhosis, 11 (22.0%) had liver failure, and 10 (20.0%) had decompensated cirrhosis at presentation. Compared to type 1, children with type 2 were younger and had higher levels of serum alanine aminotransferase [270.0 (166.0, 599.0) U/L vs. 610.0 (510.0, 1231.0) U/L, *p* = 0.003] and aspartate aminotransferase [335.0 (172.0, 756.0) U/L vs. 576.0 (433.0, 1020.0) U/L, *p* = 0.013]; however, there was no statistical significance in the outcome between the two groups (*p* > 0.05). In 48 cases that received initial immunosuppressive treatment (glucocorticoid alone = 18, in combination with azathioprine = 20, or with mycophenolate mofetil = 10), 41 (85.4%) patients survived with a native liver, and there was no statistical difference in prognosis among the three types of immunosuppressive treatments. Serum albumin levels and decompensated cirrhosis at presentation were identified as independent factors influencing the likelihood of death or the need for liver transplantation (OR 0.814 [95% CI 0.670–0.989], *p* = 0.039; OR 0.146 [95% CI 0.022–0.963], *p* = 0.046).

**Conclusion:**

The majority of paediatric AIH patients survive with a native liver, and the outcome is not related to the type of immunosuppressive therapies but to decompensated cirrhosis at presentation.

## Highlights

- There are no significant differences between type 1 and type 2 in the clinical profile of AIH in Chinese children.
- Most of paediatric AIH survive with native liver and no difference in outcome is found among different immunosuppressive therapies.
- Decompensated cirrhosis at presentation was identified as independent factors for death or liver transplantation in paediatric AIH.

## Introduction

Autoimmune hepatitis (AIH) is an immune-mediated chronic liver disease that, if untreated, usually causes cirrhosis, liver failure, and death (1). AIH in childhood has been increasingly reported (2, 3). Its incidence varies from 0.23 to 0.4/100,000 per year, and its prevalence varies from 2.4 to 9.9/100,000 people (1, 4). The incidence of AIH among Taiwanese children hospitalized with hepatitis is 2.3% (5). In South Korea, the prevalence rates of AIH in children aged 0–9 and 10–19 were 0.08 and 0.47 per 100,000 persons during 2009–2013, respectively (6). Emerging evidence indicates that HLA-DRB alleles play a significant pathogenic role in adult and paediatric AIH (7–10). The possession of HLA-DRB1*03, HLA-DRB1*07, and HLA-DRB1*13 was reported to be related to more severe liver injury (8). In addition to hepatic morbidity, the quality of life in children is significantly impaired worse than in adults, leading to the long-term physical, psychosocial, and treatment-related burden borne by children and their families (11).

Paediatric AIH is conventionally classified as type 1 or 2 based on the autoantibody detection pattern (1). According to standard knowledge, type 2 is believed to be more severe and has higher relapse rates than type 1 (3). However, recent studies have shown that the clinical, biochemical, histological, and prognostic features of AIH in both adults and children are independent of autoantibody status, calling into question the prognostic significance of AIH type according to autoantibodies (3, 12, 13). The first-line therapy for paediatric AIH consists of an initial dose of prednisolone (0.5–1 mg/kg/day) with subsequent tapering combined with or without azathioprine (AZA) (1). Since some children experience AZA intolerance or adverse effects, mycophenolate mofetil (MMF) has been gradually recommended to treat paediatric AIH (1).

The clinical characteristics and outcomes of AIH vary with the ethnic background (14, 15). The majority of current data on paediatric AIH are largely based on studies from Caucasian populations (2, 12, 16, 17). In China, clinical researches on AIH are also mostly based on adult data, while there have been limited studies on paediatric AIH (18, 19). Therefore, this study attempts to add to the limited available research on paediatric AIH through a retrospective investigation of the clinical features of paediatric AIH cases from a single Chinese centre, especially the differences between types 1 and 2 and outcomes among different immunosuppressive treatments (ISTs).

## Materials and methods

### Research design and ethics

The data of all children diagnosed with AIH at the Fifth Medical Center of the Chinese PLA General Hospital from 1 January 2015 to 1 January 2025 were retrospectively collected. This study was approved by the Ethics Committees of the Fifth Medical Center of the Chinese PLA General Hospital (no. KY-2025-6-130-1). This retrospective study was granted exemption from informed consent for all children involved and was conducted in accordance with the 1964 Helsinki Declaration.

### Diagnostic criteria and data collection

AIH was diagnosed based on the simplified 2008 scoring criteria according to the AIH guidelines (1). All cases were sorted into two groups according to the presence of anti-nuclear antibodies (ANA), anti-smooth muscle antibodies (ASMA), and/or anti-soluble liver antigen antibodies (anti-SLA) (type 1), or of anti-liver-kidney microsome type 1 antibodies (anti-LKM1) and/or anti-liver cytosol antibodies (anti-LC) (type 2) (1, 20). ANA, ASMA, anti-mitochondrial antibodies (AMA), and anti-neutrophil cytoplasmic antibodies (p-ANCA) were detected by indirect immunofluorescence testing (Euroimmun, Lübeck, Germany), while anti-SLA, anti-LKM1, and anti-LC were tested by immunoblot testing (Euroimmun, Lübeck, Germany). Positive ANA, ASMA, AMA, and p-ANCA were considered at a titre of ≥ 1:320, ≥ 1:100, ≥ 1:100, and ≥ 1:10, respectively. Anti-SLA, anti-LKM1, and anti-LC antibodies were considered positive if the band was dyed strongly according to the standard control strip (21). The diagnosis of cirrhosis was confirmed by histological techniques (reticular fibre and Masson’s trichrome stain) and/or imaging tests (ultrasound and/or CT) (22, 23). Decompensated cirrhosis was defined as the presence of one or more significant clinical events related to cirrhosis, i.e., oesophageal varices, ascites, hepatic encephalopathy (HE), or hepatorenal syndrome (24). Paediatric ALF was diagnosed with an international normalized ratio (INR) > 1.5 with HE or >2.0 with or without HE in a previously healthy child (25). Remission was defined as normalization of serum transaminase and immunoglobulin G (IgG) levels (26, 27). Relapse was defined as an increase in ALT level ≥ 3 × ULN for children who achieved normalization (1, 28).

**Figure 1:**
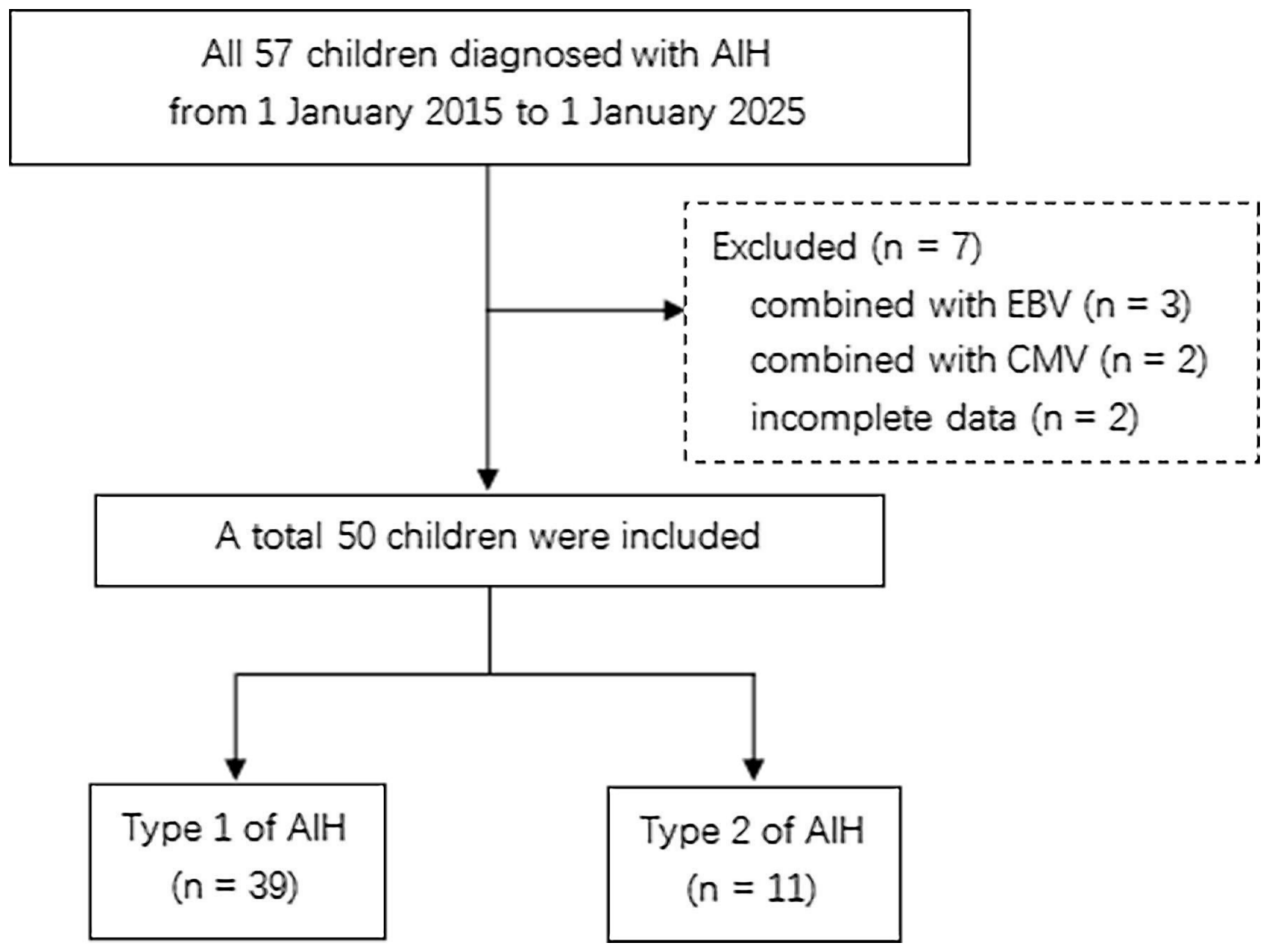
Flowchart for children with AIH.

The inclusion criteria were all children diagnosed with AIH and the exclusion of other liver diseases, such as hepatitis B, C, and E; Wilson’s disease; alpha-1-antitrypsin deficiency; non-alcoholic steatohepatitis; drug-induced liver disease; primary biliary cholangitis; and primary sclerosing cholangitis. Exclusion criteria were Epstein–Barr virus (EBV) (*n* = 3), cytomegalovirus (CMV) (*n* = 2), and incomplete data (*n* = 2) (Figure 1). Demographic data, clinical features, biochemical findings, diagnostic imaging, and liver biopsy reports were obtained from the hospital information system.

### Statistical analysis

All statistical analyses were performed using the Statistical Package for the Social Sciences (SPSS) version 26.0. Continuous variables are presented as mean ± standard deviation (SD), and categorical variables are expressed as median and interquartile range (IQR). For continuous variables, distribution was assessed using the Shapiro–Wilk test, and once the hypothesis of normality was accepted (*p* > 0.05), an independent-samples Student’s *t*-test was used for comparison between two groups. If there was no normal distribution, continuous variables were analysed using the Mann–Whitney *U*-test. Chi-squared or Fisher’s exact tests were used for categorical variables. Univariate and multivariate analyses were conducted using binary logistic regression to assess factors that could predict death or liver transplantation. For all statistical tests, a *p*-value of < 0.05 was considered statistically significant.

## Result

### Demography and clinical features

A total of 50 children with a mean onset age of 9.2 years (range, 1 to 16 years) and a majority of females, 30 (60.0%), were diagnosed with AIH (Table 1). All cases were followed up for a median of 7.1 years (range, 1 month to 22 years). The median time between the onset of symptoms and diagnosis was 3.0 months (range, 1 week to 7 years). A total of 10 children had combined type 1 diabetes (*n* = 1), idiopathic short stature (*n* = 1), and autoimmune diseases (*n* = 8), including systemic lupus erythematosus (*n* = 4), haemolytic anaemia (*n* = 2), Hashimoto thyroiditis (*n* = 1), and inflammatory bowel disease (*n* = 1). A total of 8 (16.0%) patients showed abdominal distension at presentation, but none experienced long-term constipation, uncontrollable diarrhoea, or other severe gastrointestinal symptoms during follow-up; therefore, colonoscopy was not performed.

**Table 1:** Clinical characteristics of 50 children diagnosed with types 1 and 2 AIH.

Variables	All children, *n* = 50	Type 1, *n* = 39	Type 2, *n* = 11	*p*-value
Demographic characteristics				
Age (years) at presentation	9.22 ± 3.90	9.85 ± 3.81	7.00 ± 3.55	0.031
Female children, *n* (%)	30 (60.0%)	21 (53.8%)	9 (81.8%)	0.185
Positive family history of autoimmune diseases, *n* (%)	2 (4.0%)	2 (5.1%)	0 (0.0%)	1.000
Clinical symptoms at presentation				
Fever, *n* (%)	7 (14.0%)	6 (15.4%)	1 (9.1%)	0.969
Jaundice, *n* (%)	31 (62.0%)	24 (61.5%)	7 (63.6%)	1.000
Abdominal distension, *n* (%)	8 (16.0%)	7 (17.9%)	1 (9.1%)	0.809
The time between the onset of symptoms and diagnosis (m)	3 (1, 9)	4 (2, 8)	2 (1, 15)	0.842
Laboratory results at presentation				
Albumin (g/L)	36.5 (32.0, 40.0)	36.0 (32.0, 40.0)	37.0 (33.0, 40.0)	0.997
Alanine aminotransferase (U/L)	436.4 (204.25, 752.75)	270.0 (166.0, 599.0)	610.0 (510.0, 1231.0)	0.003
Aspartate aminotransferase (U/L)	377.0 (189.0, 868.0)	335.0 (172.0, 756.0)	576.0 (433.0, 1020.0)	0.013
Total bilirubin (μmol/L)	57.6 (23.8, 124.1)	54 (18.0, 95.6)	110.3 (27.3, 190.0)	0.186
Cholinesterase (U/L)	3877.0 (3051.5, 5419.3)	3845.0 (3000.0, 5564.0)	4363.0 (3511.0, 5371.0)	0.419
International normalized ratio	1.13 (1.04, 1.33)	1.13 (1.05, 1.36)	1.09 (0.98, 1.32)	0.167
Immunoglobulin G (g/L)	25.6 (20.7, 30.6)	26.2 (21.2, 32.3)	24.7 (18.6, 26.4)	0.131
Antibodies positive at presentation, *n* (%)				
ANA	29 (58.0%)	29 (74.4%)	0 (0.0%)	<0.001
ASMA	18 (36.0%)	18 (46.2%)	0 (0.0%)	0.004
Anti-SLA	3 (6.0%)	3 (7.7%)	0 (0.0%)	1.000
Anti-LKM1	11 (22.0%)	0 (0.0%)	11 (100.0%)	<0.001
Anti-LC	1 (2.0%)	0 (0.0%)	1 (9.1%)	0.220
AMA	4 (8.0%)	4 (10.3%)	0 (0.0%)	0.563
p-ANCA	25 (50.0%)	25 (64.1%)	0 (0.0%)	<0.001
Liver ultrasound at presentation				
Portal vein diameter (mm)	9.61 ± 1.72	9.66 ± 1.75	9.43 ± 1.70	0.692
Spleen length (mm)	115.5 (98.0, 154.3)	131.0 (92.0, 158.0)	110.0 (101.0, 113.0)	0.314
Cirrhosis at presentation, *n* (%)	23 (46.0%)	18 (46.2%)	5 (45.5%)	0.967
Decompensated cirrhosis at presentation, *n* (%)	10 (20.0%)	8 (20.5%)	2 (18.2%)	1.000
Liver failure at presentation, *n* (%)	11 (22.0%)	9 (23.1%)	2 (18.2%)	1.000

ANA, anti-nuclear antibodies; ASMA, anti-smooth muscle antibodies; anti-SLA, anti-soluble liver antigen antibodies; anti-LKM1, anti-liver-kidney microsome type 1 antibodies; anti-LC, anti-liver cytosol antibodies; AMA, anti-mitochondrial antibodies; p-ANCA, anti-neutrophil cytoplasmic antibodies.

A total of 39 (78.0%) children had type 1, while the other 11 (22.0%) children had type 2. The results of autoantibodies in the 50 children at diagnosis are shown in Supplementary Table 1. Only children with type 1 AIH presented with positive AMA (10.3%) and p-ANCA (64.1%) (Table 1). The frequencies and total numbers of peripheral CD4^+^ and CD8^+^ T cells and CD19^+^ B cells at the time of diagnosis are shown in Supplementary Table 2. A total of 24 (48.0%) children had cirrhosis at the time of AIH diagnosis, 11 (22.0%) had liver failure (Supplementary Table 3), and 10 (20.0%) had decompensated cirrhosis in children with oesophageal varices (*n* = 8), ascites (*n* = 7), or hepatic encephalopathy (*n* = 3) (Supplementary Table 4). Children with type 1 diabetes were older and had lower levels of alanine aminotransferase (ALT) and aspartate aminotransferase (AST) at presentation (*p* < 0.05) (Table 1). There were no differences in sex, clinical symptoms, albumin, total bilirubin, cholinesterase, INR, IgG levels, and the prevalence of cirrhosis, decompensated cirrhosis, and liver failure at presentation between types 1 and 2 (Table 1).

### Histological features

**Figure 2:**
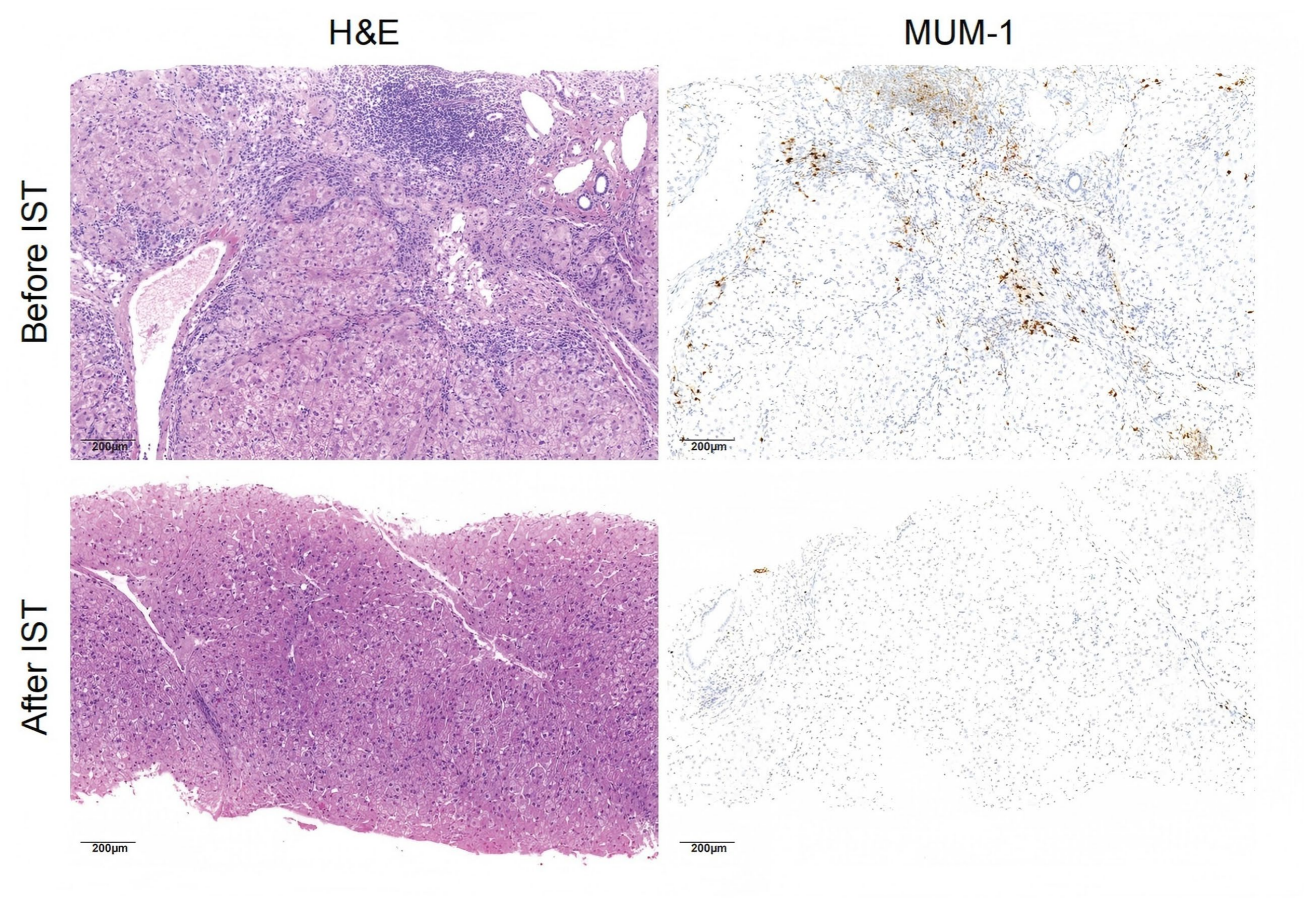
Histology of AIH in a 9-year-old male child with type 1 AIH who underwent two times of liver biopsy before and after immunosuppressive treatment (IST). In the first liver biopsy before IST, bridging necrosis, interphase hepatitis with periportal inflammation dominated by lymphocytes and emperipolesis, plasma cell infiltration predominantly located in portal tracts, hepatic rosette formation, and bile duct injury were observed. In the second liver biopsy after IST, slight interphase hepatitis with periportal inflammation, a decrease in plasma cell infiltration, and hepatic fibrosis were observed. H&E×200.

46 (92.0%) children underwent liver biopsy before IST, and the histological results are shown in Table 2. A second liver biopsy was performed in 13 children, and only 1 patient stopped IST after the second liver biopsy (Supplementary Table 5). A 9-year-old male patient who underwent two liver biopsies and went into remission had all the typical histological features of AIH, including interface hepatitis, lymphoplasmacytic infiltration, hepatocyte rosette formation, and emperipolesis (Figure 2). There was no difference in the histological features between types 1 and 2 (Table 2). 31 cases showed bile duct injuries, including bile ductular proliferation (*n* = 26), cholestasis (*n* = 5), or bile duct loss (*n* = 4). Among 23 cases of cirrhosis diagnosed by histological and/or imaging techniques, histological examination revealed bile duct injuries in 16 cases (69.6%). In 10 patients with a METAVIR score of 4, 9 (90.0%) had bile duct injuries.

**Table 2:** Histological features of 46 children at diagnosis.

Histological findings	All children with a liver biopsy, *n* = 46	Type 1 with liver biopsy, *n* = 35	Type 2 with liver biopsy, *n* = 11	*p*-value
Interface hepatitis, *n* (%)	43 (93.5%)	33 (94.3%)	10 (90.9%)	1.000
Lymphoplasmacytic infiltration, *n* (%)	45 (97.8%)	34 (97.1%)	11 (100.0%)	1.000
Hepatocyte rosette formation, *n* (%)	4 (8.7%)	3 (8.6%)	1 (9.1%)	1.000
Emperipolesis, *n* (%)	1 (2.2%)	1 (2.9%)	0 (0.0%)	1.000
Bile duct injury^*^, *n* (%)	31 (67.4%)	23 (65.7%)	8 (72.7%)	0.949
Hepatic inflammation^†^, *n* (%)				0.146
Grade 2	11 (23.9%)	7 (20.0%)	4 (36.4%)	
Grade 3	28 (60.9%)	24 (68.6%)	4 (36.4%)	
Grade 4	7 (15.2%)	4 (11.4%)	3 (27.3%)	
Hepatic fibrosis^†^, *n* (%)				0.664
Score 1	7 (15.2%)	5 (14.3%)	2 (18.2%)	
Score 2	15 (32.6%)	11 (31.4%)	4 (36.4%)	
Score 3	12 (26.1%)	9 (25.7%)	3 (27.3%)	
Score 4	10 (21.7%)	9 (25.7%)	1 (9.1%)	

*Bile duct injury includes bile ductular proliferation, cholestasis, or bile duct loss. ^†^ According to the METAVIR scoring system.

### Treatment

Of the 50 children, 48 (96.0%) received an initial IST, including glucocorticoid alone (*n* = 18) or in combination with AZA (*n* = 20) or MMF (*n* = 10). Balance 2 patients rejected the IST because their parents were afraid of its side effects. In the initial IST with the median of total course of 48.0 months (range, 0.5 to 168.0 months), prednisolone was initiated at a median dose of 1.0 mg/kg/d (range, 0.4 to 2.5 mg/kg/d), gradually decreased over a median period of 3.0 months (range, 1 to 24 months) to obtain the normalization of ALT, AST, and IgG, and finally to a median maintenance dose of 5 mg/d (1–10 mg/d). AZA was added at a dose of 50–100 mg/d, while MMF was added at a dose of 1 g/d. In one case, AZA was changed to MMF due to AZA-related thrombocytopenia. Maintenance treatment consisted of corticosteroids in combination with AZA (*n* = 17), MMF (*n* = 12), corticosteroids alone (*n* = 6), AZA alone (*n* = 3), or MMF alone (*n* = 1).

**Figure 3:**
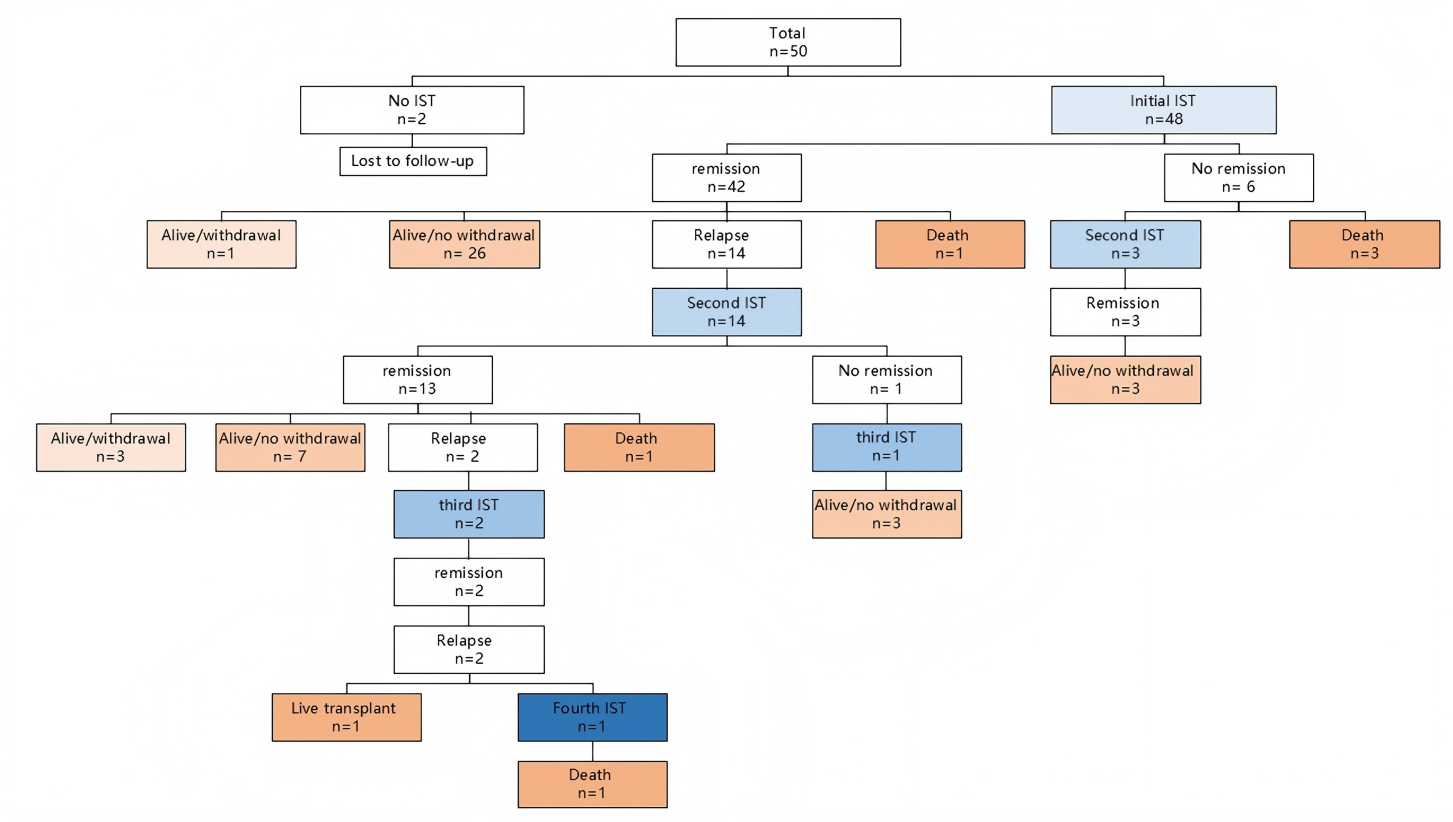
Flowchart of treatment and outcomes of the 50 children with AIH. IST, immunosuppressive treatment.

Due to relapse and no remission, 17 (34.0%) children received 2 courses of IST, 3 (6.0%) received 3 courses, and 1 (2.0%) received 4 courses (Figure 3). In cases that received 2 courses of IST, 10 (55.6%, 10/18) received glucocorticoid alone in the initial IST, 5 (25.0%, 5/20) received glucocorticoid with AZA, and 2 (20.0%, 2/10) received glucocorticoid with MMF.

### Outcome

In 48 children who received IST, the median duration of follow-up was 80.0 months (1 month to 22 years). Of these, 41 (85.4%) patients were alive with native liver, 6 (12.5%) with type 1 died, and 1 (2.1%) with type 1 underwent liver transplant after the third course of IST (Figure 3; Table 3). Children who died and underwent liver transplantation had significantly lower levels of albumin and cholinesterase and INR and a higher prevalence of cirrhosis and decompensated cirrhosis at presentation, while achieving a lower rate of remission during initial IST and a higher prevalence of multiple relapses, compared to children who were alive with native liver (*p* < 0.05) (Table 3). There was no significant difference in the frequency and total numbers of peripheral CD4^+^ and CD8^+^ T cells and CD19^+^ B cells between good (alive with native liver; *n* = 16) and bad outcomes (death or LT; *n* = 3) (Supplementary Figure 1). Only one male patient who was diagnosed with type 1 AIH was alive but did not achieve remission in an 8-year follow-up after treatment with glucocorticoid with AZA/MMF. During follow-up, three patients developed cirrhosis. In 11 cases with liver failure at presentation, 2 died, and 9 went into remission without withdrawal of the IST (Supplementary Table 3). In 10 cases with decompensated cirrhosis at presentation, 1 patient underwent liver transplant, 3 died, and 6 achieved remission without withdrawal of IST (Supplementary Table 4). The changes in autoantibodies before and after IST are presented in Supplementary Table 1. After IST, the rates of ANA, ASMA, and anti-LKM1 positivity in 48 children decreased from 60.4 to 18.8%, 37.5 to 18.8%, and 20.8 to 6.3%, respectively.

**Table 3:** Assessment of prognostic factors in paediatric AIH.

Variables	Death or liver transplant, (*n* = 7)	Alive with native liver, (*n* = 41)	*p*-value
Age (years) at presentation	10.71 ± 3.55	9.00 ± 3.91	0.284
Female, *n* (%)	5 (71.4%)	24 (58.5%)	0.687
Type 1, *n* (%)	7 (100.0%)	31 (75.6%)	0.318
Laboratory results at presentation			
Albumin (g/L)	33.0 (29.0, 35.0)	37.0 (32.5, 40.5)	0.013
Alanine aminotransferase (U/L)	153.0 (97.0, 599.0)	476.0 (213.5, 799.0)	0.077
Aspartate aminotransferase (U/L)	169.0 (106.0, 821.0)	367.0 (216.0, 807.5)	0.261
Total bilirubin (μmol/L)	60.0 (39.0, 126.0)	45.1 (17.2, 125.8)	0.350
Cholinesterase (U/L)	3275.0 (2460.0, 3845.0)	4363.0 (3223.5, 5870.5)	0.024
International normalized ratio	1.27 (1.21, 1.59)	1.11 (1.04, 1.34)	0.037
Immunoglobulin G (g/L)	28.07 (22.1, 31.6)	25.6 (20.4, 31.3)	0.517
Positive AMA	6 (85.7%)	23 (56.1%)	0.219
Positive p-ANCA	5 (71.4%)	19 (46.3%)	0.416
Cirrhosis at presentation, *n* (%)	6 (85.7%)	16 (39.0%)	0.038
Decompensated cirrhosis at presentation, *n* (%)	4 (57.1%)	6 (14.6%)	0.040
Liver failure at presentation, *n* (%)	2 (28.6%)	9 (22.0%)	1.000
Time from starting IST to remission (m)	3.0 (2.3, 10.5)	3 (1.0, 6.0)	0.625
Remission during initial IST, *n* (%)	4 (57.1%)	38 (92.7%)	0.044
Relapse after initial IST, *n* (%)	3 (42.9%)	11 (26.8%)	0.400
Underwent ≥ 2 courses of IST, *n* (%)	3 (42.9%)	14 (34.1%)	0.686
Underwent ≥ 2 times of relapse, *n* (%)	2 (28.6%)	0 (0.0%)	0.019

AMA, anti-mitochondrial antibodies; p-ANCA, anti-neutrophil cytoplasmic antibodies; IST, immunosuppression treatment.

**Figure 4:**
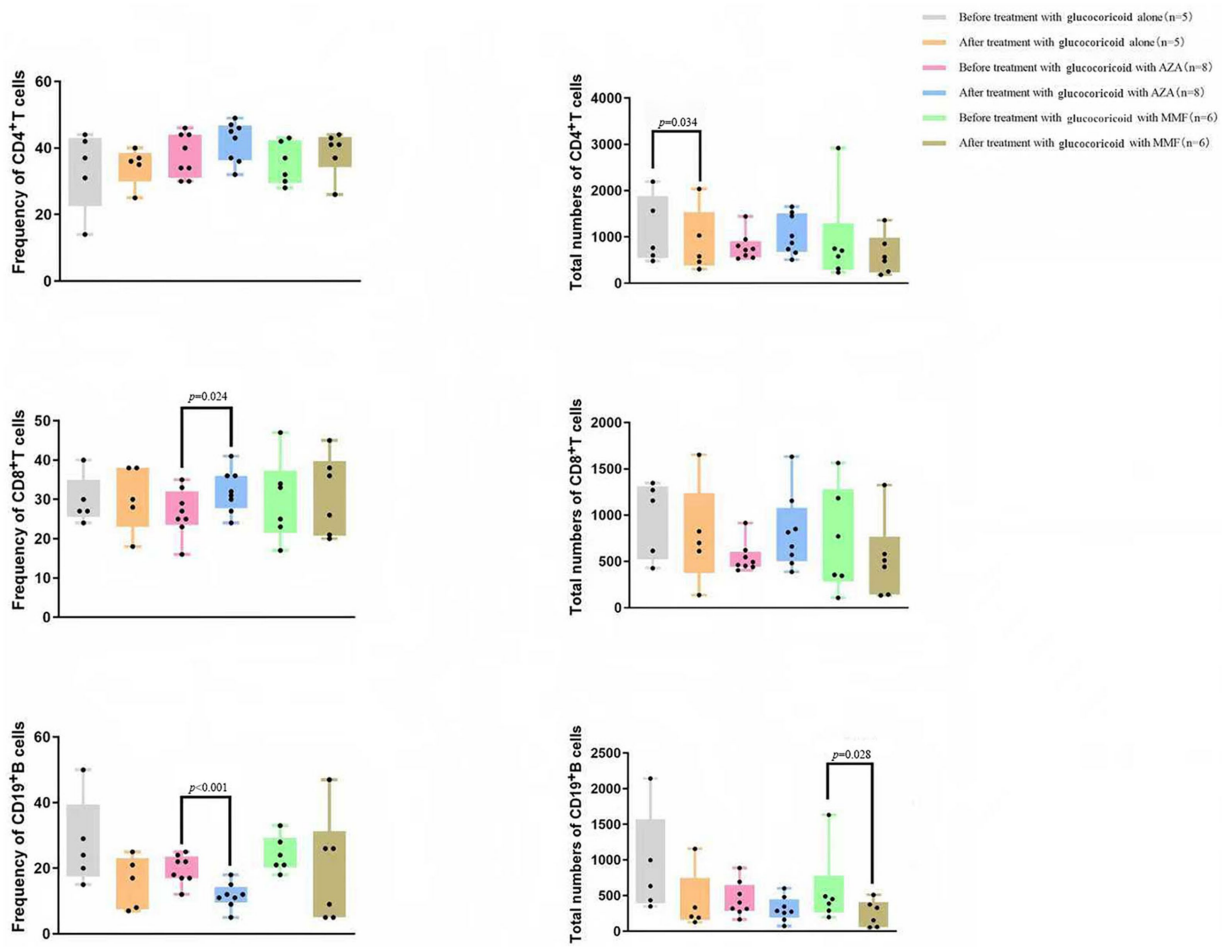
Frequency and total numbers of peripheral CD4^+^ and CD8^+^ T cells and CD19^+^ B cells before and after glucocorticoid alone, glucocorticoid with AZA, or with MMF. AZA, azathioprine; MMF, mycophenolate mofetil.

There was no detectable difference in the outcome between types 1 and 2. However, in type 1, only the proportion of children who went into remission with withdrawal of IST was lower than in type 2 (*p* = 0.025) (Table 4). Among the 3 children with type 2 who obtained remission with withdrawal, until the last follow-up in January 2025, the remission periods after cessation of IST were 41 months, 42 months, and 28 months, respectively. Only 1 patient with type 1 stopped IST after 62 months of IST and maintained sustainably normal transaminase levels during the 9-year follow-up. Among the children who received glucocorticoids alone, glucocorticoids with AZA, or glucocorticoids with MMF, there was no statistical difference in prognosis (Table 5). No significant differences were observed in the frequency or total number of peripheral CD4^+^ and CD8^+^ T cells or CD19^+^ B cells either at diagnosis or after treatment (Figure 4). Paired comparisons within each treatment group showed that the frequency of CD19^+^ B cells was significantly reduced after glucocorticoids combined with AZA, whereas the total number of CD19^+^ B cells declined after glucocorticoids combined with MMF.

**Table 4:** Treatment and follow-up of 48 children with different types of AIH who received immunosuppressive treatment.

Variables	All children received IST, *n* = 48	Type 1 received IST, *n* = 38	Type 2 received IST, *n* = 10	*p*-value
IST				0.666
Glucocorticoid alone, *n* (%)	18 (37.5%)	13 (34.2%)	5 (50.0%)	
Glucocorticoid combined with AZA, *n* (%)	20 (41.7%)	17 (44.7%)	3 (30.0%)	
Glucocorticoid combined with MMF, *n* (%)	10 (20.8%)	8 (21.1%)	2 (20.0%)	
Time of IST (m)	48.0 (13.3, 74.3)	32.0 (12.0, 72.0)	67.0 (27.0, 81.0)	0.130
Time from starting IST to remission (m)	3.0 (1.0, 6.0)	3.0 (2.0, 6.0)	1.0 (1.0, 4.5)	0.089
Remission during initial IST, *n* (%)	42 (87.5%)	33 (86.8%)	9 (90.0%)	1.000
Relapse after initial IST, *n* (%)	14 (29.2%)	9 (23.7%)	5 (50.0%)	0.216
Underwent ≥ 2 courses of IST, *n* (%)	17 (35.4%)	11 (28.9%)	6 (60.0%)	0.146
Underwent ≥ 2 times of relapse, *n* (%)	2 (4.2%)	2 (5.3%)	0 (0.0%)	1.000
Alive with native liver, *n* (%)	41 (85.4%)	31 (81.6%)	10 (100.0%)	0.318
Remission with withdrawal of IST, *n* (%)	4 (8.3%)	1 (2.6%)	3 (30.0%)	0.025
Remission without withdrawal of IST, *n* (%)	36 (75.0%)	29 (76.3%)	7 (70.0%)	1.000
No remission during IST, *n* (%)	1 (2.1%)	1 (2.6%)	0 (0.0%)	1.000
Death or liver transplantation, *n* (%)	7 (14.6%)	7 (18.4%)	0 (0.0%)	0.318

IST, immunosuppressive treatment; AZA, azathioprine; MMF, mycophenolate mofetil.

**Table 5:** Comparison of characteristics and outcomes of children according to initial immunosuppressive treatments.

Variables	Glucocorticoid alone, (*n* = 18)	Glucocorticoid with AZA, (*n* = 20)	Glucocorticoid with MMF, (*n* = 10)	*p*-value
Age (years) at presentation	9.0 (4.8, 13.0)	10.0 (7.0, 12.8)	8.5 (6.8, 12.3)	0.747
Female, *n* (%)	13 (72.2%)	8 (40.0%)	8 (80.0%)	0.044
Type 1, *n* (%)	13 (72.2%)	17 (85.0%)	8 (80.0%)	0.666
Laboratory results at presentation				
Albumin (g/L)	36.58 ± 3.96	35.76 ± 6.39	35.40 ± 7.92	0.859
Alanine aminotransferase (U/L)	539.0 (157.3, 871.3)	436.9 (211.7, 748.1)	272.5 (161.5, 546.0)	0.435
Aspartate aminotransferase (U/L)	549.5 (331.0, 899.3)	318.5 (176.3, 721.5)	281.0 (174.8, 527.5)	0.280
Total bilirubin (μmol/L)	40.4 (21.1, 130.4)	61.0 (29.3, 124.8)	40.8 (12.3, 120.3)	0.514
Cholinesterase (U/L)	4832.0 (3333.3, 5835.8)	3645.0 (3303.5, 4701.3)	3276.0 (2533.5, 5819.3)	0.351
International normalized ratio	1.08 (1.03, 1.27)	1.13 (1.05, 1.48)	1.27 (1.05, 1.54)	0.177
Immunoglobulin G (g/L)	25.18 ± 5.99	30.22 ± 6.60	22.01 ± 7.16	0.005
Cirrhosis at presentation, *n* (%)	8 (44.4%)	9 (45.0%)	5 (50.0%)	1.000
Decompensated cirrhosis at presentation, *n* (%)	4 (22.2%)	3 (15.0%)	3 (30.0%)	0.599
Liver failure at presentation, *n* (%)	2 (11.1%)	5 (25.0%)	4 (40.0%)	0.217
Time of IST (m)	27.0 (12.0, 72.8)	54.0 (21.0, 80.8)	36.0 (12.0, 81.8)	0.635
Time from starting IST to remission (m)	3.0 (2.5,4.5)	3.0 (1.0,6.0)	3.0 (1.0,9.0)	0.999
Remission during initial IST, *n* (%)	13 (72.2%)	19 (95.0%)	10 (100.0%)	0.081
Relapse after initial IST, *n* (%)	8 (44.4%)	4 (20.0%)	2 (20.0%)	0.211
Underwent ≥ 2 courses of IST, *n* (%)	10 (55.6%)	5 (25.0%)	2 (20.0%)	0.079
Underwent ≥ 2 times of relapse, *n* (%)	2 (11.1%)	0 (0.0%)	0 (0.0%)	0.176
Alive with native liver, *n* (%)	13 (72.2%)	19 (95.0%)	9 (90.0%)	0.123
Remission with withdrawal of IST	3 (16.7%)	1 (5.0%)	0 (0.0%)	0.412
Remission without withdrawal of IST	10 (55.6%)	17 (85.0%)	9 (90.0%)	0.072
No remission during IST	0 (0.0%)	1 (5.0%)	0 (0.0%)	1.000
Death or liver transplantation, *n* (%)	5 (27.8%)	1 (5.0%)	1 (10.0%)	0.123

IST, immunosuppressive treatment.

During IST, the side effects related to glucocorticoids were observed, including cataract (*n* = 12), growth retardation (*n* = 5), central obesity (*n* = 4), infection (*n* = 3), fracture (*n* = 1), and acne (*n* = 1). In 10 cases, pre-treatment testing of the thiopurine methyltransferase level was performed. Thrombocytopenia related to AZA occurred in two cases, while leukopenia and hair loss occurred in one case each. No case was reported with the side effect of MMF.

### Prognostic indicators

Univariate analysis was conducted to determine the predictors of good (live with native liver; *n* = 41) or bad (death or LT; *n* = 7) outcomes. On multivariate analysis, albumin and decompensated cirrhosis at presentation were found to be significant independent factors for poor outcomes (OR 0.814 [95% CI 0.670–0.989], *p* = 0.039; OR 0.146 [95% CI 0.022–0.963], *p* = 0.046) (Table 6).

**Table 6:** Binary logistic regression for predicting poor outcomes (death or LT).

	Univariate analysis		Multivariate analysis	
Variables	OR	*p*-value	OR	*p*-value
Albumin	0.815 (0.681, 0.975)	0.025	0.814 (0.670, 0.989)	0.039
Cholinesterase	0.999 (0.998, 1.000)	0.050		
International normalized ratio	13.687 (0.562, 333.404)	0.108		
Cirrhosis at presentation	0.107 (0.012, 0.970)	0.047		
Decompensated cirrhosis at presentation	0.129 (0.023, 0.725)	0.020	0.146 (0.022, 0.963)	0.046

## Discussion

This study is one of the few researches on AIH in the Asian population, particularly in children (14, 29, 30). In China, while the data on paediatric AIH remain limited, studies suggest that AIH is not rare, according to reports (31, 32). Cao et al. reported that in Chinese children, the majority of cases have progressive liver fibrosis at the diagnosis of AIH, and long-term IST is essential due to inevitable relapse after IST withdrawal (19). In other Asian countries, Tsuyoshi et al. first studied the clinical profile of paediatric AIH in 35 Japanese children in a nationwide survey, including a high positive rate of HLA-DR4, low prevalence of cirrhosis, and high prevalence of type 1 (33). Lee et al. (30) reported the clinical features of paediatric AIH in 32 Malaysian children and found that these are similar to those reported in Caucasian children. Low et al. (34) investigated the clinical features and outcomes in 10 children with AIH and found that AIH was an important cause of liver disease in Singapore. In India, paediatric AIH generally presented with advanced hepatic disease, but the majority survived with native liver after IST was administered (35).

In this study, similarities and differences were found between paediatric AIH in China and in other countries. Female sex and type 1 predominance of paediatric AIH in this study are similar to those reported in other Asian and European studies (17, 30, 33). As reported, the insidious and non-specific clinical symptoms in this study were variable, such as fever, jaundice, and abdominal distension, which may lead to a prolonged time to diagnosis by months (16, 30). Considering the confusing diagnosis and different treatments for AIH and hepatitis caused by non-hepatotropic viruses, this study excluded cases combined with EBV or CMV infection, even though all of the cases presented typical histological features of AIH and survived after IST following antiviral therapy. The proportion of children with cirrhosis (48%) and liver failure (22%) at presentation was similar to that reported previously (17, 30). However, the rate of decompensated cirrhosis (20%) at presentation was lower than that in Indian children (42.9%) (35). In China, due to increasing recognition of paediatric AIH and adequate access to healthcare, AIH is diagnosed at an early stage, and IST is initiated before the decompensated cirrhosis stages to decrease fibrosis progression and lead to a lower prevalence of decompensated cirrhosis (23, 31, 36).

In this study, a high ratio (92.0%) of children underwent liver biopsy to confirm the diagnosis of AIH, and the results showed that interface hepatitis and lymphoplasmacytic infiltration were typical lesions, while hepatocyte rosette formation and emperipolesis were not specific for AIH and are no longer considered typical lesions of AIH in the 2025 EASL guidelines on AIH (1). In addition, bile duct injuries, including bile ductular proliferation, cholestasis, and bile duct loss, were commonly detected in this study, with the exclusion of PBC or PSC by liver biopsy or MRCP, indicating that the therapeutic addition of ursodeoxycholic acid to standard IST could be helpful (1, 37).

In this study, the clinical characteristics, pathology, and prognosis of type 1 or type 2 AIH were compared to provide some confirmation of standard knowledge and challenge some accepted ideas on paediatric AIH. Similar to Malaysian (16.0%) and Indian (18.6%) studies, the proportion of type 2, which is positive for anti-LKM1 or anti-LC, in this study was 22.0%, whereas type 2 is reported to be rare in Japan and Singapore (30, 33–35). The frequency of p-ACNA in adults has been reported to range from 40 to 96% in type 1 (38, 39). In this study, positive p-ANCA was only present in type 1 children, significantly different from that in type 2 children, suggesting that p-ANCA may also be an important marker of type 1 in children. In the 2015 EASL guidelines on AIH, it was claimed that more frequent failure of IST occurs in type 2 cases than in type 1 cases (40). However, a recent large long-term observational study on paediatric AIH showed that the prognosis of AIH was independent of autoantibody status (12). In this study, children with type 2 disease had higher levels of serum ALT and AST at presentation than those with type 1, but no statistical significance was observed in the majority of the clinical characteristics, histological features, and outcomes. This might be related to the fact that elevated levels of aminotransferases do not reliably reflect the histological severity of AIH (40). Thus, in 2025 updated EASL guidelines on AIH, sub-classification of AIH according to autoantibodies are questionable and remain an ongoing debate (1).

The treatment for paediatric AIH is corticosteroids alone or in combination with AZA or MMF (1). In this study, 62.5% of the patients received glucocorticoids in combination with AZA or MMF, while 37.5% received corticosteroids alone. The main maintenance treatment for AIH is low-dose corticosteroids combined with AZA or MMF. Combination therapy with a low dose of corticosteroid is correspondingly associated with fewer corticosteroid-related side effects, such as cataracts and growth retardation, than glucocorticoid monotherapy, which is of utmost importance in children (1, 41, 42). Combination therapy has been reported to have similar efficacy to glucocorticoid monotherapy in terms of outcome, and survival with native liver is not different between glucocorticoids in combination with AZA and MMF (43–45). In this study, there was also no significant difference in the prognosis of AIH among corticosteroid alone, in combination with AZA, and with MMF, indicating that the combination therapy aims to minimize exposure to corticosteroids but might not achieve better outcomes. However, because this was a retrospective study with limited cases and a follow-up period, these results should be interpreted with caution. Interestingly, a recent publication discovered that IST could deplete regulatory B cells, which play an important role in the maintenance of tolerance and immune homeostasis, and this inhibitory effect persisted throughout IST (46). In this study, after IST, a reduction in the frequency and total number of CD19^+^ B cells in children was also observed; however, further research is needed to elucidate the role of B cells and their long-term implications in AIH treatment.

The majority of paediatric AIH, including liver failure, responds well to IST, obtains clinical and biochemical remission, and survives with native liver, but a few children still die or need liver transplantation (1, 16, 31, 47). In this study, 14.6% of patients had poor outcomes, while 18.2% of patients with liver failure died. Thus, based on the clinical presentation and stage of AIH at diagnosis, it is important to identify factors that influence survival. Cirrhosis or decompensation at presentation are adverse prognostic parameters to predict mortality (37, 48). In this study, among the factors linked to poor outcomes (death or liver transplant), the highest associations were the high proportion of decompensated cirrhosis and the low level of albumin as one of the clinical manifestations of decompensated cirrhosis, but not lymphocyte subsets, the type of AIH, the existence of liver failure, or different immunosuppressive therapies. Thus, children with decompensated cirrhosis at diagnosis should be managed and evaluated for liver transplantation (1). In addition, in terms of response to IST, children with remission during initial IST tend to have a good outcome (alive with native liver), while those children who have multiple relapses are more likely to die or require liver transplantation (28, 49).

Recent publications have demonstrated that HLA-DRB alleles have clinical and immunological implications in AIH (7–10). Ma et al. (8) reported that in children of European ancestry, HLA-DRB1*03 predisposed them to type 1 of AIH and HLA-DRB1*07 to type 2, HLA-DRB1*03 or HLA-DRB1*13 was associated with fibrosis at disease onset, and possession of these three genes was related to severe liver damage. Wang et al. (10) found a link between AIH-predisposing genes (HLA DR3/DR7/DR13) and defective immunoregulation (low frequencies of T regulatory cells) both in children and their first-degree relatives with ethnicities of Caucasian, Black, Indian, and Arab. In China, further studies should be carried out on the link between HLA-DRB alleles and outcomes of paediatric AIH, comparing with findings from adults or different ethnic groups, in order to expand the relatively scarce data on the association of HLA-DRB allele profile with AIH in Chinese children.

This study was observational research from a single centre, so the incidence and prevalence of AIH in China could not be estimated from the present study. Children who test negative for antibodies may have been missed. Finally, as this was a retrospective study with a restricted number of study subjects for subgroup comparisons, the results should be interpreted cautiously.

In conclusion, this study provides a chance to understand the clinical profile of AIH in Chinese children. The results showed no significant differences between types 1 and 2. Survival with the native liver was achieved in the majority of Chinese children with AIH, and no difference in outcomes was found among prednisolone alone, in combination with AZA, and with MMF. Decompensated cirrhosis at presentation adversely affects the outcome of paediatric AIH in China.

## Data Availability

The original contributions presented in the study are included in the article/Supplementary material, further inquiries can be directed to the corresponding authors.

## Glossary

AIH: autoimmune hepatitis
AZA: azathioprine
MMF: mycophenolate mofetil
ANA: anti-nuclear antibodies
ASMA: anti-smooth muscle antibodies
anti-SLA: anti-soluble liver antigen antibodies
anti-LKM1: anti-liver-kidney microsome type 1 antibodies
anti-LC: anti-liver cytosol antibodies
HE: hepatic encephalopathy
INR: international normalized ratio
IgG: immunoglobulin G
EBV: Epstein–Barr virus
CMV: cytomegalovirus
SD: standard deviation
IQR: interquartile range
ALT: alanine aminotransferase
AST: aspartate aminotransferase
IST: initial immunosuppressive treatment
AMA: anti-mitochondrial antibodies
p-ANCA: anti-neutrophil cytoplasmic antibodies
LT: live transplant
PBC: primary biliary cholangitis
PSC: primary sclerosing cholangitis
MRCP: magnetic resonance cholangiopancreatography.
