# Supplementary material for: Clinical characteristics and outcomes in 50 children with autoimmune hepatitis: a retrospective study from a single centre in China
Source: Front Med (Lausanne). 2026 Jan 12;12:1733006. doi: 10.3389/fmed.2025.1733006 (PMC12833044; doi:10.3389/fmed.2025.1733006)
Supplement: Supplementary file 1 [file Data_Sheet_1.pdf]

Supplementary table 1. Results of autoantibodies in 50 children before and after immunosuppressive treatment.

	Type	Immunosuppressive treatments	Before treatment					After treatment				
			ANA	SMA	SLA	Anti-LKM-1	Anti-LC	ANA	SMA	SLA	Anti-LKM-1	Anti-LC
1	1	Glucocorticoid alone	1:320	1:100	Negative	Negative	Negative	1:100	Negative	Negative	Negative	Negative
2	1	Glucocorticoid alone	1:320	Negative	Negative	Negative	Negative	1:100	Negative	Negative	Negative	Negative
3	1	Glucocorticoid alone	1:320	1:320	Negative	Negative	Negative	Negative	1:100	Negative	Negative	Negative
4	1	Glucocorticoid alone	> 1:1000	Negative	Negative	Negative	Negative	1:100	Negative	Negative	Negative	Negative
5	1	Glucocorticoid alone	1:320	> 1:1000	Negative	Negative	Negative	1:100	> 1:1000	Negative	Negative	Negative
6	1	Glucocorticoid alone	> 1:1000	Negative	Negative	Negative	Negative	> 1:1000	Negative	Negative	Negative	Negative
7	1	Glucocorticoid alone	Negative	Negative	+++	Negative	Negative	Negative	Negative	+++	Negative	Negative
8	1	Glucocorticoid alone	1:320	Negative	Negative	Negative	Negative	Negative	Negative	Negative	Negative	Negative
9	1	Glucocorticoid alone	Negative	Negative	+++	Negative	Negative	Negative	Negative	+++	Negative	Negative
10	1	Glucocorticoid alone	1:320	1:100	Negative	Negative	Negative	Negative	Negative	Negative	Negative	Negative
11	1	Glucocorticoid alone	1:320	Negative	Negative	Negative	Negative	Negative	Negative	Negative	Negative	Negative
12	1	Glucocorticoid alone	1:1000	Negative	Negative	Negative	Negative	1:320	Negative	Negative	Negative	Negative
13	1	Glucocorticoid alone	Negative	1:1000	Negative	Negative	Negative	Negative	1:320	Negative	Negative	Negative
14	1	Glucocorticoid with AZA	1:1000	1:1000	Negative	Negative	Negative	1:100	Negative	Negative	Negative	Negative
15	1	Glucocorticoid with AZA	1:1000	Negative	Negative	Negative	Negative	1:320	Negative	Negative	Negative	Negative
16	1	Glucocorticoid with AZA	1:320	Negative	Negative	Negative	Negative	1:100	Negative	Negative	Negative	Negative
17	1	Glucocorticoid with AZA	1:320	1:100	Negative	Negative	Negative	Negative	1:100	Negative	Negative	Negative
18	1	Glucocorticoid with AZA	1:320	1:320	Negative	Negative	Negative	Negative	1:320	Negative	Negative	Negative
19	1	Glucocorticoid with AZA	1:320	Negative	Negative	Negative	Negative	1:100	Negative	Negative	Negative	Negative

20	1	Glucocorticoid with AZA	1:1000	Negative	Negative	Negative	Negative	1:320	Negative	Negative	Negative	Negative
21	1	Glucocorticoid with AZA	1:320	Negative	Negative	Negative	Negative	1:320	Negative	Negative	Negative	Negative
22	1	Glucocorticoid with AZA	Negative	1:320	Negative	Negative	Negative	Negative	1:100	Negative	Negative	Negative
23	1	Glucocorticoid with AZA	1:1000	1:100	Negative	Negative	Negative	Negative	Negative	Negative	Negative	Negative
24	1	Glucocorticoid with AZA	1:320	Negative	Negative	Negative	Negative	1:100	Negative	Negative	Negative	Negative
25	1	Glucocorticoid with AZA	1:1000	1:100	Negative	Negative	Negative	1:320	Negative	Negative	Negative	Negative
26	1	Glucocorticoid with AZA	Negative	1:320	Negative	Negative	Negative	Negative	Negative	Negative	Negative	Negative
27	1	Glucocorticoid with AZA	1:1000	Negative	Negative	Negative	Negative	1:320	Negative	Negative	Negative	Negative
28	1	Glucocorticoid with AZA	1:1000	1:320	Negative	Negative	Negative	1:1000	1:320	Negative	Negative	Negative
29	1	Glucocorticoid with AZA	Negative	1:320	Negative	Negative	Negative	Negative	1:320	Negative	Negative	Negative
30	1	Glucocorticoid with AZA	1:320	1:1000	Negative	Negative	Negative	1:100	1:100	Negative	Negative	Negative
31	1	Glucocorticoid with MMF	1:1000	Negative	Negative	Negative	Negative	1:1000	Negative	Negative	Negative	Negative
32	1	Glucocorticoid with MMF	1:320	Negative	Negative	Negative	Negative	Negative	Negative	Negative	Negative	Negative
33	1	Glucocorticoid with MMF	Negative	Negative	+++	Negative	Negative	Negative	Negative	+++	Negative	Negative
34	1	Glucocorticoid with MMF	1:320	Negative	Negative	Negative	Negative	1:100	Negative	Negative	Negative	Negative
35	1	Glucocorticoid with MMF	1:320	Negative	Negative	Negative	Negative	1:100	Negative	Negative	Negative	Negative
36	1	Glucocorticoid with MMF	Negative	1:100	Negative	Negative	Negative	Negative	Negative	Negative	Negative	Negative
37	1	Glucocorticoid with MMF	> 1:1000	Negative	Negative	Negative	Negative	1:100	Negative	Negative	Negative	Negative
38	1	Glucocorticoid with MMF	Negative	1:100	Negative	Negative	Negative	Negative	Negative	Negative	Negative	Negative
39	2	Glucocorticoid alone	Negative	Negative	Negative	+++	Negative	Negative	Negative	Negative	+++	Negative
40	2	Glucocorticoid alone	Negative	Negative	Negative	+++	Negative	Negative	Negative	Negative	+++	Negative

41	2	Glucocorticoid alone	Negative	Negative	Negative	+++	Negative	Negative	Negative	Negative	Negative	Negative
42	2	Glucocorticoid alone	Negative	Negative	Negative	+++	Negative	Negative	Negative	Negative	Negative	Negative
43	2	Glucocorticoid alone	Negative	Negative	Negative	+++	Negative	Negative	Negative	Negative	Negative	Negative
44	2	Glucocorticoid with AZA	Negative	Negative	Negative	++	+++	Negative	Negative	Negative	Negative	++
45	2	Glucocorticoid with AZA	Negative	Negative	Negative	+++	Negative	Negative	Negative	Negative	+++	Negative
46	2	Glucocorticoid with AZA	Negative	Negative	Negative	+	Negative	Negative	Negative	Negative	Negative	Negative
47	2	Glucocorticoid with MMF	Negative	Negative	Negative	+++	Negative	Negative	Negative	Negative	+++	Negative
48	2	Glucocorticoid with MMF	Negative	Negative	Negative	+++	Negative	Negative	Negative	Negative	Negative	Negative
49	1	Reject IST	Negative	1:320	Negative	Negative	Negative	-	-	-	-	-
50	2	Reject IST	Negative	Negative	Negative	+++	Negative	-	-	-	-	-

Supplementary table 2. Frequency and total numbers of peripheral CD4+ and CD8+ T cells and CD19+ B cells in 19 cases.

	Type	Immunosuppressive treatments	Prognosis	Before treatment						After treatment					
				$CD4^+$ T cells		$CD8^+$ T cells		$CD19^+$ B cells		$CD4^+$ T cells		$CD8^+$ T cells		$CD19^+$ B cells	
				Frequency, %	Count, /uL	Frequency, %	Count, /uL	Frequency, %	Count, /uL	Frequency, %	Count, /uL	Frequency, %	Count, /uL	Frequency, %	Count, /uL
1	1	Glucocorticoid alone	Alive with native liver	37	1567	30	1271	15	635	35	1033	28	826	7	207
2	1	Glucocorticoid alone	Liver transplant	42	764	24	428	24	437	36	580	38	612	8	129
3	1	Glucocorticoid alone	Death	31	480	40	616	29	348	40	304	18	137	25	190
4	1	Glucocorticoid with AZA	Alive with native liver	30	534	35	623	18	320	32	509	36	572	18	286
5	1	Glucocorticoid with AZA	Alive with native liver	44	717	27	440	17	277	37	873	36	850	11	260
6	1	Glucocorticoid with AZA	Alive with native liver	34	809	23	547	22	524	46	660	27	387	12	164
7	1	Glucocorticoid with AZA	Alive with native liver	34	551	25	405	25	405	36	1451	41	1632	12	481
8	1	Glucocorticoid with AZA	Alive with native liver	30	948	29	916	22	695	47	1022	31	663	11	277
9	1	Glucocorticoid with AZA	Alive with native liver	46	1444	16	494	24	888	45	1533	24	815	15	604
10	1	Glucocorticoid with MMF	Alive with native liver	28	706	47	1184	18	454	44	569	45	581	5	61
11	1	Glucocorticoid with MMF	Alive with native liver	43	2923	23	1564	24	1632	43	854	26	512	26	512
12	1	Glucocorticoid with MMF	Alive with native liver	37	235	17	107	33	388	26	186	20	142	47	375
13	1	Glucocorticoid with MMF	Death	30	315	34	356	28	199	41	256	21	134	26	155

14	2	Glucocorticoid alone	Alive with native liver	14	601	27	1158	50	2145	25	466	38	701	17	334
15	2	Glucocorticoid alone	Alive with native liver	44	2196	27	1347	20	998	37	2039	30	1653	21	1157
16	2	Glucocorticoid with AZA	Alive with native liver	40	740	25	463	17	315	43	1656	30	1155	9	347
17	2	Glucocorticoid with AZA	Alive with native liver	44	605	33	453	12	165	49	737	32	482	5	74
18	2	Glucocorticoid with MMF	Alive with native liver	42	580	25	345	21	290	41	483	38	442	5	56
19	2	Glucocorticoid with MMF	Alive with native liver	32	749	33	772	21	491	37	1362	36	1325	9	331

Supplementary table 3. Clinical characteristics and outcome of 11 children diagnosed with liver failure of AIH.

	Gender	Age (y)	Type	Clinical symptoms	Time between the onset of symptoms and diagnosis (m)	Albumin (g/L)	ALT (U/L)	AST (U/L)	Total bilirubin (μmol/L)	Cholin esteras e (U/L)	INR	IgG (g/L)	Positive auto-antibodies	Cirrhosis	Decomp ensated cirrhosis	Initial IST	Time of IST (m)	Time from starting IST to remission (m)	Outcome
1	Female	10	1	Jaundice	0.5	31.4	1442	1703	377	3000	1.52	28.7	ANA	No	No	Glucocor ticoid alone	12	3	Remission without withdrawal of IST
2	Female	10	1	Jaundice	0.5	35	373	1176	228	1810	1.76	28.07	ANA	No	No	Glucocor ticoid alone	0.5	No remission	Death
3	Female	13	1	Jaundice	3	20.9	153	169	84.9	3274	1.59	26.1	ANA	Yes	No	Glucocor ticoid with AZA	10	2	Death
4	Male	6	1	Jaundice	0.3	32	1009	895	385.7	2750	1.72	30.3	ANA	No	No	Glucocor ticoid with AZA	120	3	Remission without withdrawal of IST
5	Female	16	1	/	0.2	31	250	302	28.2	2393	1.67	33.47	ANA, SMA	Yes	No	Glucocor ticoid with AZA	108	3	Remission without withdrawal of IST
6	Male	9	1	Epistaxis	8	33	561	618	43.8	3392	1.52	32.28	SMA	Yes	No	Glucocor ticoid with AZA	62	6	Remission without withdrawal of IST
7	Male	13	1	Jaundice and epistaxis	84	28	33	129	42.6	2558	1.53	33.83	ANA	Yes	No	Glucocor ticoid with MMF	87	1	Remission without withdrawal of IST
8	Male	12	1	Fever, jaundice,	0.8	26	188	244	63.6	1680	1.74	34.43	ANA	Yes	Yes	Glucocor ticoid	13	12	Remission without

				and abdominal distension												with MMF			withdrawal of IST
9	Female	7	1	Jaundice	8	35	576	1066	241.1	3041	1.51	18.79	ANA	No	No	Glucocorticoid with MMF	96	3	Remission without withdrawal of IST
10	Male	8	2	Jaundice	1	32	476	576	326	3600	1.52	26.42	Anti-LKM-1	No	No	Glucocorticoid with AZA	56	1	Remission without withdrawal of IST
11	Female	9	2	Jaundice	3	34	1760	1552	110.3	3511	1.55	24.27	Anti-LKM-1	No	No	Glucocorticoid with MMF	66	6	Remission without withdrawal of IST

ALT, Alanine aminotransferase; AST, Aspartate aminotransferase; INR, International normalized ratio; IgG, Immunoglobulin G.

Supplementary table 4. Clinical characteristics and outcome of 10 children diagnosed with decompensated cirrhosis of AIH.

	Gender	Age (y)	Type	Clinical symptoms	Time between the onset of symptoms and diagnosis (m)	Albumin (g/L)	ALT (U/L)	AST (U/L)	Total bilirubin (μmol/L)	Cholinesterase (U/L)	INR	IgG (g/L)	Positive auto-antibodies	Liver failure	Initial IST	Time of IST (m)	Time from starting IST to remission (m)	Outcome
1	Female	1	2	Abdominal distension	84	34	1231	1020	27.3	5371	0.99	18.79	Anti-LKM-1	No	Glucocorticoid alone	28	6	Remission with withdrawal of IST
2	Male	7	1	Jaundice, abdominal distension	9	36	676	619	34.2	3845	1.27	30.3	ANA, SMA	No	Glucocorticoid alone	12	3	Death or liver transplantation
3	Female	10	1	Jaundice	0.7	33	100	106	57.6	3909	1.32	31.63	ANA	No	Glucocorticoid alone	18	3	Death or liver transplantation
4	Female	12	1	Abdominal distension	0.2	30	97	160	60	3426	1.1	22.1	ANA, SMA	No	Glucocorticoid alone	26	/	Death or liver transplantation
5	Male	8	1	Jaundice, abdominal distension	5	43.4	794	335	95.6	2096	1.13	32.67	ANA	No	Glucocorticoid with AZA	71	6	Remission without withdrawal of IST,
6	Female	8	1	Jaundice,	2	33.6	397.	366.	57.6	4238	1.1	38.7	ANA	No	Glucocorticoid	28	/	Remission without

				abdominal distension			8	1							with AZA			withdrawal of IST,
7	Male	13	1	Jaundice	5	37	270	231	128	3499	1.26	24.82	ANA	No	Glucocorticoid with AZA	9	3	Remission without withdrawal of IST,
8	Female	9	2	Jaundice	3	34	1760	1552	110.3	3511	1.55	24.27	Anti-LKM-1	Yes	Glucocorticoid with MMF	66	6	Remission without withdrawal of IST
9	Female	16	1	Jaundice, abdominal distension	1	29	82	87	39	2460	1.21	18.01	Anti-SLA	No	Glucocorticoid with MMF	24	13	Death or liver transplantation
10	Male	12	1	Fever, jaundice, and abdominal distension	0.8	26	188	244	63.6	1680	1.74	34.43	ANA	Yes	Glucocorticoid with MMF	13	12	Remission without withdrawal of IST

ALT, Alanine aminotransferase; AST, Aspartate aminotransferase; INR, International normalized ratio; IgG, Immunoglobulin G.

Supplementary table 5. Clinical and histological features of 13 AIH children performed two times of liver biopsy.

	Gender	Age(y)	Type	Initial IST	First pathologic findings	Second Pathologic findings	Stopped IST after the second liver biopsy
1	Female	4	1	Glucocorticoid alone	G3S2	G1S1	Yes
2	Female	3	2	Glucocorticoid alone	G2S3	G3S2	No
3	Female	7	1	Glucocorticoid alone	G2S1	G1S1	No
4	Female	4	1	Glucocorticoid alone	G3S4	G1S4	No
5	Male	4	1	Glucocorticoid alone	G3S3	G2S1	No
6	Male	8	1	Glucocorticoid with AZA	G3S3	G4S3	No
7	Female	8	1	Glucocorticoid with AZA	G3S2	G2S1	No
8	Male	9	1	Glucocorticoid with AZA	G3S2	G2S2	No
9	Male	6	1	Glucocorticoid with AZA	G3S2	G1S1	No
10	Female	10	1	Glucocorticoid with AZA	G4S2	G1S3	No
11	Male	5	1	Glucocorticoid with AZA	G2S1	G1S0	No
12	Male	9	1	Glucocorticoid with AZA	G4S3	G1S4	No
13	Female	8	2	Glucocorticoid with MMF	G4S4	G1S3	No

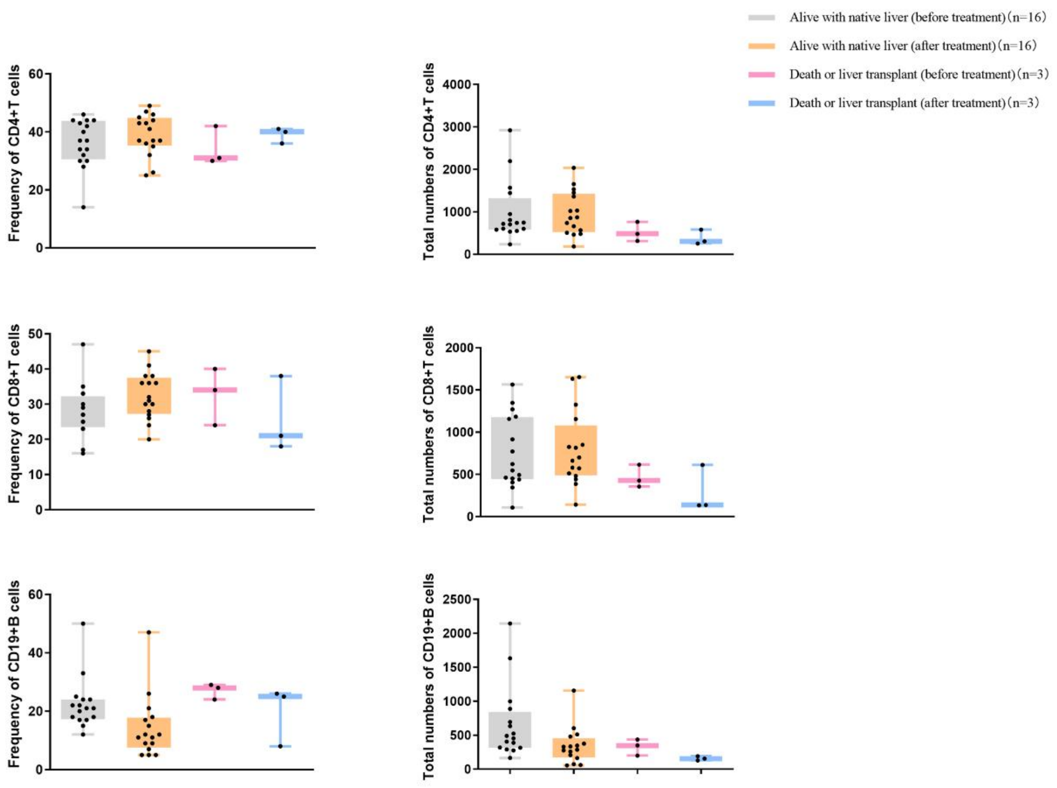

Supplementary figure 1. Frequency and total numbers of peripheral CD4^{+} and CD8^{+} T cells and CD19^{+} B cells between good (alive with native liver; n = 16) and bad outcome (death or LT; n = 3).
