# Supplementary figures and images for: Clinical characteristics and outcomes in 50 children with autoimmune hepatitis: a retrospective study from a single centre in China
Source: Front Med (Lausanne). 2026 Jan 12;12:1733006. doi: 10.3389/fmed.2025.1733006 (PMC12833044; doi:10.3389/fmed.2025.1733006)

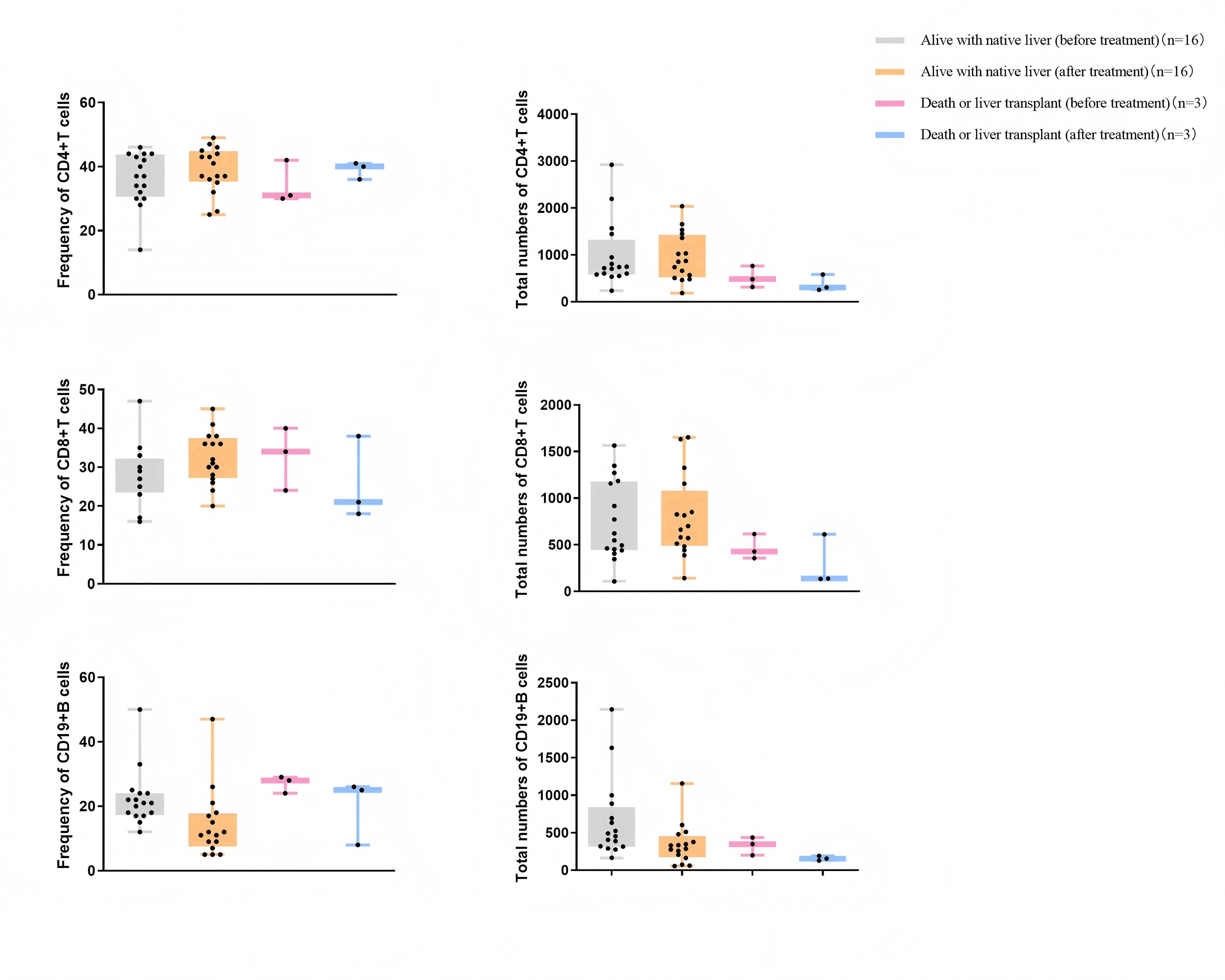

Supplement: Supplementary file 2 [file Image_1.jpeg]
